# Supplementary material for: Identification of photoperiod-induced specific miRNAs in the adrenal glands of Sunite sheep (Ovis aries)
Source: Front Vet Sci. 2022 Jul 22;9:888207. doi: 10.3389/fvets.2022.888207 (PMC9354845; doi:10.3389/fvets.2022.888207)
Supplement: Supplementary file 1 [file Data_Sheet_1.ZIP › Supplementary file/miRDeep2 file.zip!/miRDeep2 file/Novel_Structure/SP42A2/Novel_502.pdf]

| | | |
|---|---|---|
| Provisional ID | : | NC_019484.2_43668 |
| Score total | : | 1.6 |
| Score for star read(s) | : | -1.3 |
| Score for read counts | : | 0 |
| Score for mfe | : | 1.3 |
| Score for randfold | : | 1.6 |
| Score for cons. seed | : | |
| Total read count | : | 22 |
| Mature read count | : | 22 |
| Loop read count | : | 0 |
| Star read count | : | 0 |

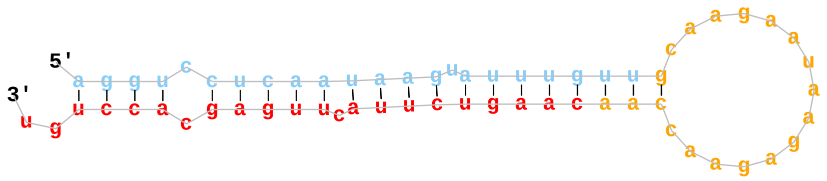


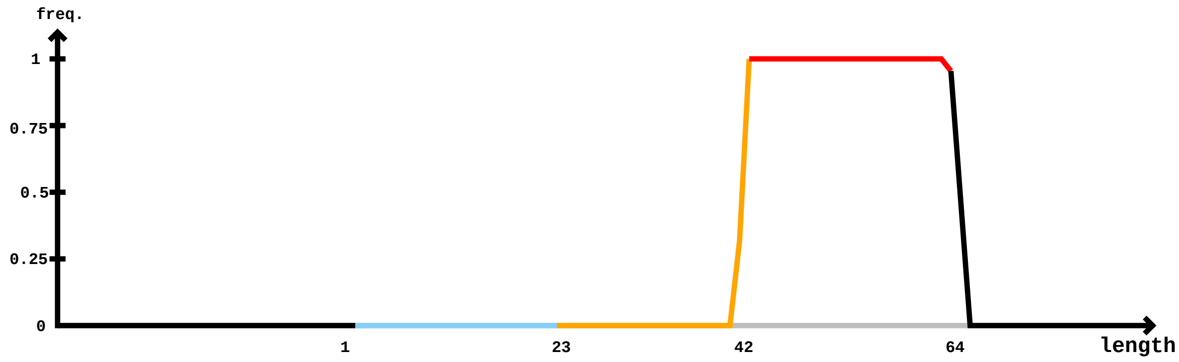


```
                          Star                                     Mature
5'- auuguguuucuucaugccugacauauagcagguccucaauaaguauuuguugcaagaauaagagaaccaacaagucuuacuugagcaccuguuauguggaggauaaaaagcu   -3'   exp
    .....((((.((.((.(((..((((((((((((.(((((((((.((((((((...............)))))))))))).))))).)))))))))))).))))))).)))).   reads   mm   sample
    .....................................................................acaagucuuacuugagcaccugu....................   5       0    seq
    .....................................................................acaagucuuacuugagcaccuguu...................   2       0    seq
    ......................................................................caagucuuacuugagcaccug.....................   1       0    seq
    ......................................................................caagucuuacuugagcaccugu....................   13      0    seq
    ......................................................................caagucuuacuugagcaccuguu...................   1       0    seq
```
